# Neural mechanisms of credit card spending

**DOI:** 10.1038/s41598-021-83488-3

**Published:** 2021-02-18

**Authors:** Sachin Banker, Derek Dunfield, Alex Huang, Drazen Prelec

**Affiliations:** 1 Eccles School of Business, University of Utah, Salt Lake City, UT 84112 USA; 2 MIT Sloan Neuroeconomics Laboratory, Massachusetts Institute of Technology, Cambridge, MA 02142 USA; 3 Sloan School of Management, Massachusetts Institute of Technology, Cambridge, MA 02142 USA; 4 Department of Brain and Cognitive Sciences, Massachusetts Institute of Technology, Cambridge, MA 02142 USA; 5 Department of Economics, Massachusetts Institute of Technology, Cambridge, MA 02142 USA

**Keywords:** Neuroscience, Human behaviour

## Abstract

Credit cards have often been blamed for consumer overspending and for the growth in household debt. Indeed, laboratory studies of purchase behavior have shown that credit cards can facilitate spending in ways that are difficult to justify on purely financial grounds. However, the psychological mechanisms behind this spending facilitation effect remain conjectural. A leading hypothesis is that credit cards reduce the pain of payment and so ‘release the brakes’ that hold expenditures in check. Alternatively, credit cards could provide a ‘step on the gas,’ increasing motivation to spend. Here we present the first evidence of differences in brain activation in the presence of real credit and cash purchase opportunities. In an fMRI shopping task, participants purchased items tailored to their interests, either by using a personal credit card or their own cash. Credit card purchases were associated with strong activation in the striatum, which coincided with onset of the credit card cue and was not related to product price. In contrast, reward network activation weakly predicted cash purchases, and only among relatively cheaper items. The presence of reward network activation differences highlights the potential neural impact of novel payment instruments in stimulating spending—these fundamental reward mechanisms could be exploited by new payment methods as we transition to a purely cashless society.

## Introduction

Since their introduction in the 1960s, credit cards have gradually replaced cash and check transactions as the default payment method for consumer purchases, and are now the fastest growing method in the United States^1^. In the future credit cards may find themselves overtaken by digital wallets and other devices. From an economic perspective, it is not surprising that technological changes in payment transactions have some impact on macroeconomic variables, notably on U.S. household debt, which has been steadily rising over the last two decades^2,3^. This historical debt increase may be, in part, a rational household response to new lines of credit and to the other benefits of credit cards, in terms of convenience, security, and reward points.

However, evidence is accumulating that suggests credit cards take advantage of cognitive biases and other psychological mechanisms. Many, if not most consumers overestimate their future ability to repay and are surprised by the high interest charges when these come due^4–6^. Empirical studies show that shoppers with credit cards are willing to spend more on items^7,8^, check out with bigger baskets^9^, focus on and remember more product benefits rather than costs^10,11^, and make more indulgent and unplanned purchase choices^12,13^.

Do credit cards then serve to “release the brakes” on spending or instead act to “step on the gas”? Prior evidence indicates that, in fact, both mechanisms may be involved, such that spending facilitation effects are likely to be driven by combination of these processes. For instance, most relevant to this paper are reports that mere exposure to credit card logos can stimulate spending^14–16^. As first argued by Feinberg^14^, spending facilitation via mere exposure implicates classical conditioning mechanisms and cue-triggered cravings associated with addiction^17,18^. Salient cues can often trigger a motivational urge to pursue its reward, such as the pleasure associated with consumption—in this way fueling greater spending.

Yet, recent literature has focused greater attention upon an alternative mechanism derived from the mental accounting literature. That is, credit cards may instead weaken brakes on spending by lessening the pain associated with making payments. This “pain-of-payment” hypothesis was originally proposed in a metaphorical sense^19,20^, however more literal interpretations have also taken root more recently. With credit card purchases, the act of payment is temporally removed from the act of acquisition, and is further decoupled when multiple transactions, perhaps spread over many months, are represented as a single consolidated balance. This dissociation of purchasing from payment may put costs out of mind and reduce the influence of price on product purchase decisions.

Understanding the brain mechanisms that are responsible for these effects is important, as they are not likely to be confined to credit cards only. By tapping these mechanisms, any new payment technology can disturb old expenditure patterns in ways that people fail to anticipate, and may come to regret.

In this exploratory study, we provide the first evidence of differences in brain activation in the presence of real credit and cash purchase opportunities, presented in an fMRI shopping task. Participants used their own personal credit card or cash funds to make real purchases of products while we simultaneously observed brain activity. Our study focuses on the purchase of everyday products with cash and credit at relatively small dollar values, similar to those examined within prior literature on payment methods. We find that activation in the classical reward networks (the striatum) differentiates credit card purchases from non-purchases, and, importantly, bears little relation to price. In contrast, activation in these same networks is a weak predictor of cash purchases, but interacts with price to predict purchases of cheaper instead of more expensive items. Activation in the insula, a brain region previously linked to pain-of-paying^21–24^, does not differentiate credit from cash purchases in our study.

As we discuss in the conclusion, we cannot rule out that a reduction in pain-of-paying is responsible for credit card overspending at higher dollar amounts than those used in the study. However, our results suggest that classical cue-conditioning and the resulting sensitization of neural reward networks may have a separate role in motivating credit card purchases. Even if credit cards do “release the brakes” on spending, as argued by mental accounting, it appears that they could also help to “step on the gas.”

To facilitate comparisons with previous results, our study builds on the established SHOP (“Save Holdings Or Purchase”) fMRI paradigm^21,22,25^. In the task, participants make a series of purchase decisions for products offered at a steep discount relative to market price. A trial begins with a screenshot of a product that the participant has not seen previously in the study, followed by the product price, and concludes with a “buy” versus “no-buy” decision screen. Neural signals in the task have been shown to dissociate reward-related from price-related decision processing^21^. For this reason, it is a natural protocol for assessing competing hypotheses about credit card purchase facilitation mechanisms.

**Figure 1:**
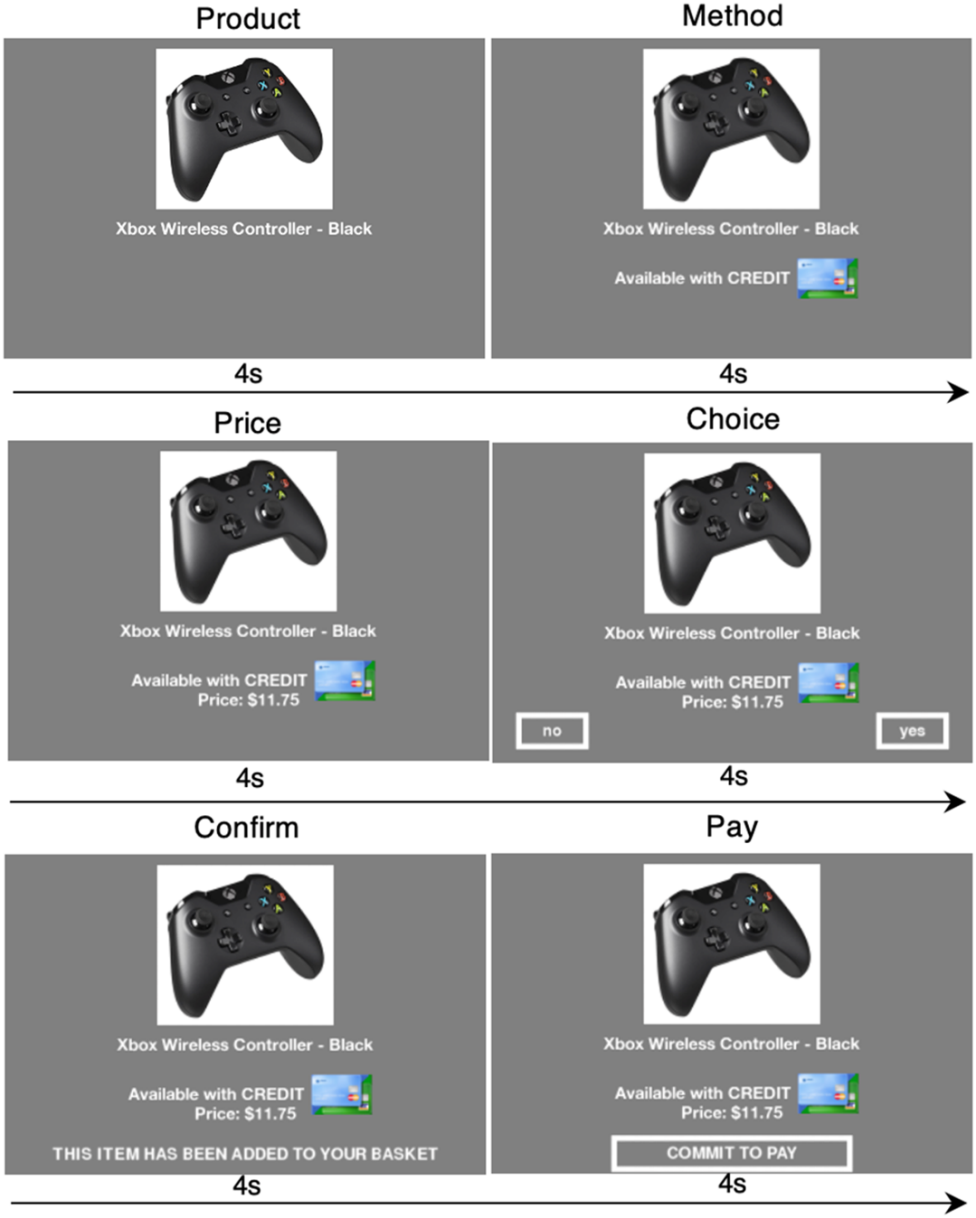
Shopping task trial structure in the current study. Participants viewed the product for 4 s, the payment method for 4 s, the price for 4 s, and then made a choice to purchase within 4 s. Post-decisional periods consisted of a confirmation, 4 s, and a pay response, 4 s. Purchase trial shown; if not purchased, the confirmation indicated “basket unchanged” and the pay screen indicated “no payment necessary.” Intertrial interval jittered 2–8 s. This study added the Method, Confirm, and Pay phases to the original SHOP paradigm.

**Figure 2:**
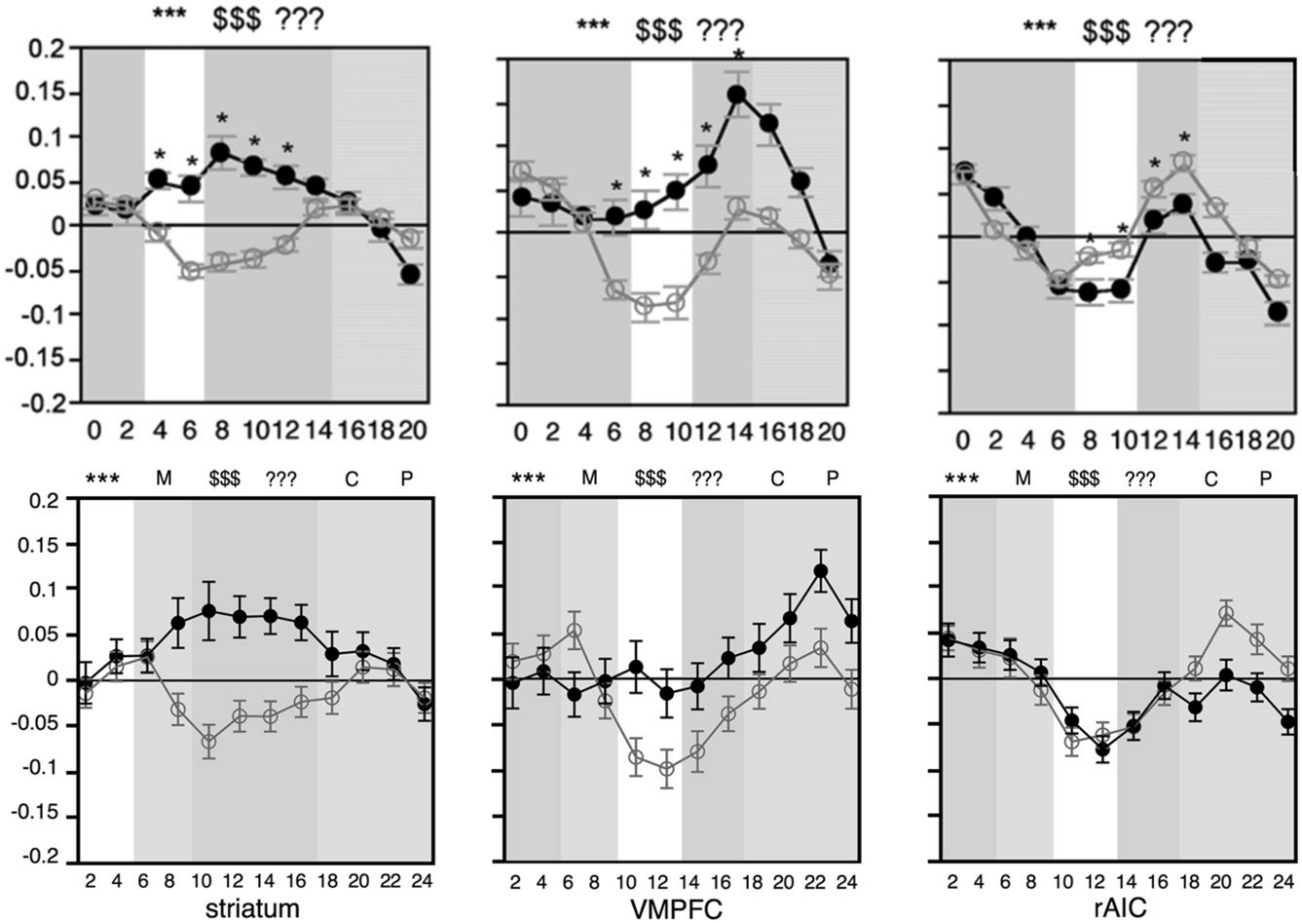
Comparison with Knutson et al.^21^. Neural activation time courses in the striatum, VMPFC, and rAIC distinguishing purchase (black) from non-purchase (grey), y-axis labeled with percent signal change. Above: Fig. 2 from Knutson et al.^21^. Below: time courses from the current study collapsed across payment methods. Phases: * = product, M = method, $ = price, ? = choice, C = confirm, P = pay.

In the SHOP task, a decision to buy is marked by three neural signals (see Fig. 1 for SHOP trial structure; Fig. 2 top panel for activation pattern in the original SHOP study). Two signals come from the classic dopaminergic reward network involving the striatum and the ventromedial prefrontal cortex (VMPFC). Striatal activation is a leading predictor of purchase, appearing during the product and price screens, but losing significance by the decision point. Activation in the VMPFC predicts purchase during the price presentation and decision points, and also correlates with post-scanner estimates of consumer surplus (defined as the difference between stated willingness-to-pay for a product and its price). Thus, VMPFC activity has been interpreted as a net-value signal within a range of decision making contexts^21,22,25–28^.

A separate neural indicator of product purchase is reduced activity in the right anterior insula cortex (rAIC), when the price appears^21,22,25^. Because the rAIC has been previously implicated in the processing of negative emotions and pain^29–34^, its activation in the SHOP task has been interpreted as evidence consistent with a “pain-of-paying” caused by high price, acting as a brake on spending^21,22^. Paying for products has been thought to elicit an affective pain experience associated with activation in the anterior portion of the right insular cortex, in contrast to the posterior portion of the right insular cortex which has been linked to representation of physical pain experiences^23^.

## Results

### Behavioral findings

The independent variables, product price and payment method, had the expected effects on purchase behavior in the fMRI shopping task. A hierarchical logistic regression predicting purchase decisions yielded parameters on price (*b* = − 0.334, *se* = 0.116, *p* = 0.004), payment method (*b* = − 0.036, *se* = 0.099, *p* = 0.715) and their interaction (*b* = 0.251, *se* = 0.120, *p* = 0.037) in the anticipated direction. This interaction follows predictions based on a prior test conducted in a similar context^35^. Consistent with previous empirical studies^7,8^, participants were more willing to purchase higher-price items with credit rather than with cash, and thus they spent more overall when using credit card (average basket = $87.41, *SD* = 61) rather than cash ($84.19, *SD* = 51). These behavioral findings supported the notion that credit cards facilitate purchasing behavior, and our analysis presented below focuses primarily on the associated neural activation evidence.

### Neural activation

**Figure 3:**
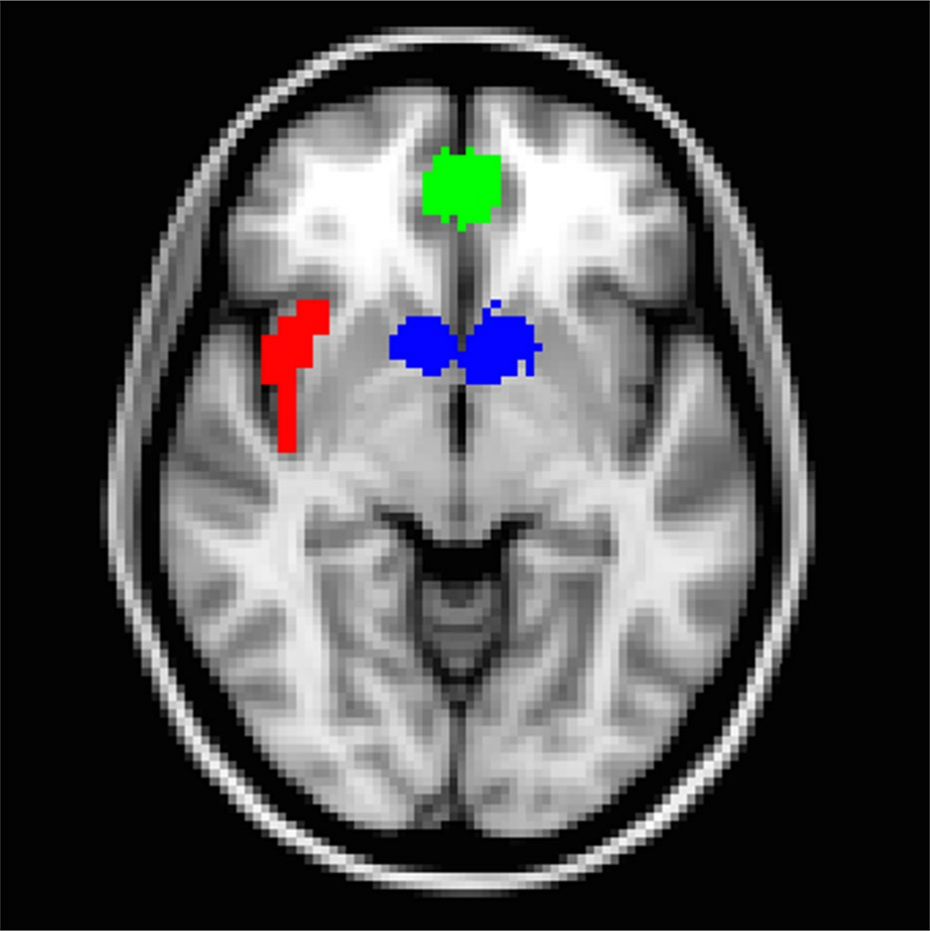
Regions of interest examined within the current study. Ventromedial prefrontal cortex shown in green and striatum shown in blue, from Bartra et al.^26^ meta-analysis; right anterior insular cortex (rAIC) shown in red, from Kelly et al.^34^ parcellation analysis; MNI x = − 6, y = 10, z = − 6.

The current fMRI design follows the approach in the original SHOP article^21^. For comparison, Fig. 2 displays the activation time course in the current study alongside the Knutson et al.^21^ results. Collapsing across payment methods, the time course in each region of interest (ROI) tracks the original results to a remarkable degree. The key regions of interest—striatum, VMPFC, and rAIC—are shown graphically within Fig. 3. While we do not consider the current findings to be an exact replication of the original SHOP results, the neural activation patterns from the earlier study provide a benchmark reference, as discussed below.

**Figure 4:**
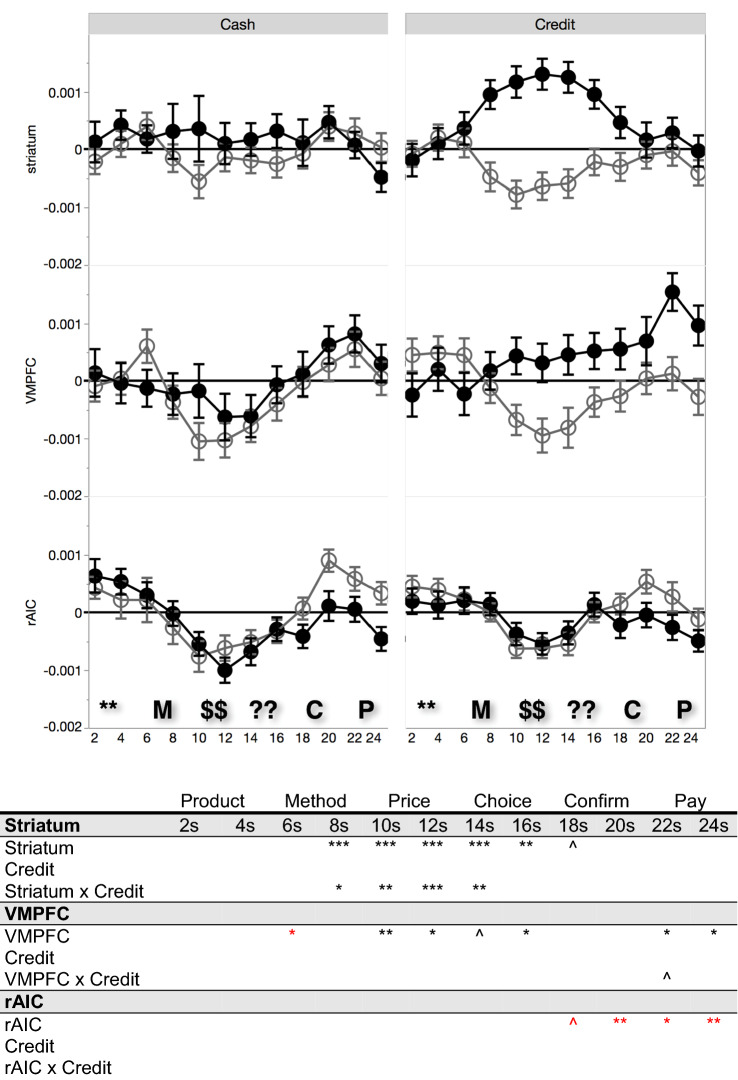
Above: ROI signal intensity time courses illustrating purchase (black) versus non-purchase (grey). Below: Buy decision regressed on ROI signal intensity, payment method, and interaction at each TR. Red indicates a negative coefficient. Parameter significance denoted by ***p < .001, **p < .01, *p < .05, ^*p* < .10. Phases: * = product, M = method, $ = price, ? = choice, C = confirm, P = pay.

Figure 4 breaks apart the time courses by payment method, and shows that the reward network differential buy signal is clearly present with credit card purchases, but is negligible with cash purchases. Logistic regressions of the purchase decision on the ROI signal change, payment method, and their interaction confirms that credit purchases were associated with greater differential striatal activation, beginning with the payment method screen and extending up until the decision screen (shown in the bottom panel of Fig. 4). The same pattern holds directionally but not significantly, for the VMPFC. However, if the neural signal in each ROI is collapsed across buy and no-buy decisions, there is no significant difference between credit card and cash trials, at any time point, suggesting that presentation of the credit card stimulus per se does not affect brain activity in the target ROIs.

Looking at the cash trials only, the reward signals are weaker predictors of purchases than in the original SHOP task^21^, even though the earlier study also required cash payments. However, in Knutson et al.^21^, participants tapped their experimental endowment—money they did not have before the study—potentially creating a house-money effect. In contrast, participants in the current study paid out-of-pocket with the $50 in cash they brought to the experiment.

As evident in Fig. 2, collapsing across payment methods in the current study reveals activation time courses that track the Knutson et al.^21^ results. This appears to be primarily due to purchase decisions using a credit card, not cash. Accordingly, comparing the current findings to past SHOP results indicates that credit card purchase decisions resemble house-money purchases. Thus, one interpretation of these findings is that when shopping with credit card, individuals act as if drawing on an endowment (from the financial institution backing the card).

**Figure 5:**
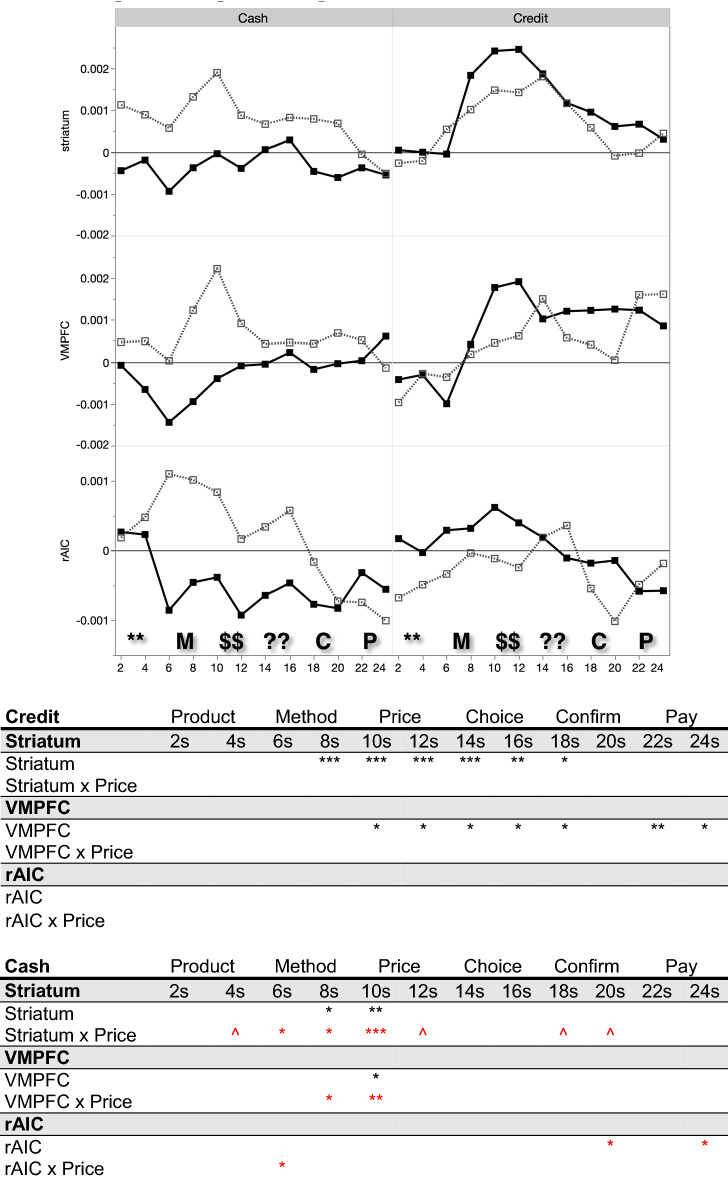
Above: y-axis plots the difference between average purchase and average non-purchase ROI signal intensity, for high-price (black) and low-price (grey) items by payment method. Below: Buy decision regressed on ROI signal intensity and the interaction between price (continuous) and ROI signal intensity at each TR, separately for credit and for cash. Red indicates a negative coefficient. Parameter significance denoted by ***p < .001, **p < .01, *p < .05, ^*p* < .10.

An additional analysis shows that prices modulate the association of neural signals and the decision to purchase. The y-axis in Fig. 5 displays the differential purchasing signal. That is, we take the average ROI activation on purchase trials and the average ROI activation on non-purchase trials, and plot the difference between these means; this is plotted separately for high-price items and for low-price items, when using cash and when using credit (see Figure S2 in Supplementary Information for further information). Accordingly, points plotted at the zero line indicate that neural activation did not differ between purchase and non-purchase decisions on average. Points plotted above the zero line instead indicate that purchases were associated with greater activation in the ROI relative to non-purchase decisions (and conversely for points below the zero line). The significance levels in the table in Fig. 5 come from logistic regressions of the buy decision on ROI signal change and its interaction of signal with item price (a continuous variable).

Focusing on decisions using cash, positive reward-related ROI activation in the striatum was associated with purchasing only among lower-priced items, and this differential purchasing signal is near zero for higher-priced items (see the left panel in Fig. 5). Confirmed by the interactions in the regression analysis, buying items with cash has a price-dependent neural signature that is clearest in the striatum. In contrast, the neural signature associated with credit purchases is not price-contingent, and is instead reflected by differential activation in reward-related ROIs, regardless of the price. Regression analyses that directly compare the differential sensitivity to price when using cash and credit are reported within the Supplementary Information; these findings suggest that credit cards reduce sensitivity to price information via heightened striatal activation, exhibited during the periods in which product price is presented to participants.

## Discussion

Although a single experiment is rarely definitive with respect to behavior outside of the lab, the results reported here provide clear clues about the neural mechanisms that differentiate credit card from cash purchases and that may be implicated in credit card overspending.

A leading hypothesis within recent literature is that credit cards facilitate purchasing by diminishing a pain-of-payment that would otherwise keep spending in check. The intuition behind it is that card transactions “decouple” (disassociate) payments from consumption^19,20^. The decoupling occurs because the payment is delayed, can be postponed repeatedly, and the actual repayment date may be ambiguous if diverse expenditures are lumped into a rolling balance. Decoupling of payments from consumption allows people to keep the cost of the item “out of mind,” creating a kind of analgesic at the moment of purchase.

We do not find neural evidence for this explanation, at least if pain is defined as a physical sensation and insula activity treated as its neural marker, as has been suggested in the past^21–23^. Although insular activation does differentiate purchase from non-purchase decisions, it does so only after the decision point, and does not clearly interact with either payment method or item price (Figs. 4, 5). Insular activation seems to reflect simple product rejection in our study, perhaps similar to the rejection of bad offers in economic games^36–38^. Yet, our evidence is consistent with the more metaphorical interpretation of the pain-of-payment account. That is, while we did not observe credit cards to influence pain processing networks in the brain, our evidence did indicate that price information failed to have any modulating influence on neural mechanisms associated with credit card purchases (i.e., costs were out of mind).

At the same time, there are a number of important constraints within the current study that offer worthy directions for further exploration. For instance, it is possible that spending cash could elicit stronger negative affective responses at higher price levels than those examined within the current study. Some interesting exploratory research suggests that observing others make cash payments at higher price levels is associated with increased activation in the insula^24^. As applied within prior literature, our study design also mimics typical retail shopping environments in which participants add items to their basket and subsequently checkout (rather than parting with money at the moment the purchase decision is made) which may diminish the salience of cash payments. Furthermore, in conveying the payment method to participants, we also used an icon that included both Visa and Mastercard logos; additional research could help to clarify the role of brand logos in eliciting spending facilitation effects. As participants in this study had reasonable levels of financial literacy, additional research focusing on consumers with lower, or higher, levels of financial literacy and experience would be valuable to pursue. Additionally, while our study aimed to stick closely to prior SHOP tasks, more highly powered designs could offer greater insight into the role of the insula.

Taken altogether, the hypothesis that gains most support from the current evidence is that the reward network—the striatum in particular—has been chronically sensitized by prior experience with credit cards. In line with cue-triggered accounts of cravings, exposure to conditioned credit card cues may trigger sensitivity to rewards^14,17,18,39,40^. Such sensitization would show up in a reward anticipation increase following onset of the credit card logo in expectation of an imminent buy decision, a pattern that we indeed observe within striatal activity. Under this hypothesis, credit card cues may in part activate the pursuit of rewarding products rather than merely alleviating the pain associated with paying for them.

The difference in reward network activation between credit and cash conditions is notable in light of the small prices and modest behavioral effects. Self-reports taken after the shopping task suggest that participants were largely unaware of the influence of payment methods on their decisions, disagreeing with statements that they were more impulsive and less price-conscious when shopping with credit cards (see Supplementary Information). The differences in reward-related neural purchasing signals observed between payment methods do not appear to reflect inconveniences in using cash itself; indeed, prior SHOP studies examining cash purchases^21^ documented similar reward-related neural purchasing signals, so long as participants were spending house-money from an experimental endowment. Further research could help to clarify the extent to which consumers consider shopping with credit card to be akin to spending house money. We do find that the impact of credit cards on behavior (purchase likelihood) and neural activity increases with price. Extrapolating on this price-related trend, one might expect greater credit card effects for big-ticket items in an actual marketplace.

Although recent literature largely interprets credit card facilitation of spending through a pain-of-payment lens, a considerable body of existing behavioral evidence is consistent with a cue-triggered account. Findings that credit card cues serve to heighten attention and memory toward the positive elements and away from the negative elements of product stimuli^10,11,41^ fall in line with conditioning processes that have long been understood at both psychological and neurobiological levels^42,43^. Exposure to credit card logos has also been shown to increase the willingness to pay for items even when people pay with cash^14–16^, consistent with the idea that credit cards can serve as cues that trigger spending behavior. Moreover, while traditional mental accounting theories suggest that credit cards lessen pain-of-paying for all types of products, people are in fact more inclined to purchase vice products when shopping with credit cards^12^, as is suggested by an account in which credit cards prompt the pursuit of products that satisfy cue-triggered cravings. A conditioned spending response can lead individuals to become more attuned to consumption cues and also raise the marginal utility of consumption^39,40^.

Behavioral economic models with expectation-based reference points could potentially accommodate our findings and allow analytical extrapolation from the lab to the marketplace. The general idea in these models is that experience with a transaction instrument generates expectations, which then serve as a reference point^39,40,44^. If the expectations are to purchase, then failing to purchase becomes a loss relative to the reference point. Such models have explained addictive behavior in the past, however the expectation formation could be localized to a combination of card, product category, and physical environment (e.g., retail or online). In principle, any distinct transaction method: cash, credit, check or digital wallet, could stamp in its own unique set of “local preferences,” as the consumer accumulates experience.

It is notable that the neural mechanisms involved in facilitating credit card spending share similarities to neural mechanisms that have in the past been implicated in addictive behaviors. Specifically, our evidence indicates that credit card cues led to reward network sensitization in the striatum, a distinguishing feature of cue-triggered mechanisms that has emerged in studies of chemical addiction to substances^17,18,45^. While we certainly do not claim that consumers are “addicted” to credit cards, an appreciation of the overlapping physical substrates may offer insights into important individual differences in vulnerabilities to more extreme forms of credit card overspending. For example, the genetic factors involved in dopaminergic reward network function that have been linked to drug addiction^46^ could also contribute to greater risk of credit card abuse, due to the underlying role they play in learning and conditioning processes.

Credit cards are now an established instrument, but similar neural effects may arise with any disruptive payment technology. New payment methods and digital currencies can sensitize reward networks in unexpected ways, removing the financial guardrails created by old purchasing habits and routines. Many new payment technologies have the ability to strengthen reinforcement mechanisms through the use of unique sounds heard when acquiring an item, visual notifications received on mobile devices, and even haptic stimuli that can simultaneously provide physical feedback. Such multisensory stimuli^47^ can drive speedier conditioning in a way that could very quickly begin to impact consumer purchasing processes. Payment methods that are integrated within mobile devices could also exploit prior conditioning with the device and fuel more unrestrained purchasing behavior^48^.

This is a cautionary message for the consumer finance and payment industries, as well as for economic welfare analysis based on revealed preference. If neural mechanisms operate under the radar, one cannot assume that technical improvements in payment methods will make all consumers better off. Our study does not discuss consumer protection and related policy issues, but underlines the importance of keeping policy eyes open to neuroscience evidence as it comes in. Because novel payment methods have the potential to take advantage of the neurobiological processes that drive purchase behavior, developing guardrails to prevent misuse may enable consumers to fully benefit from advancements in payment technology.

Although payment methods are involved in every consumer purchase decision, the underlying mechanisms through which they operate have not been well understood. The current findings highlight considerable differences in brain mechanisms responsible for the influence of payment methods on purchasing decisions, and expose important consumer vulnerabilities that will require attention as payment methods rapidly evolve. Ultimately, each of the many billions of consumer financial transactions that occur across the world each year are made by individuals who share the neural mechanisms studied here.

## Methods

### Participants

A total of twenty-eight participants (ages 20–54; age *M* = 28.7, *SD* = 10.6; 18 women) completed the study. One participant was excluded from the analysis due to excessive head motion during the scan (more than 3 mm). The experimental procedures were approved by the MIT Institutional Review Board and were performed in accordance with relevant guidelines and regulations. All participants provided informed consent. Participants were compensated at least $75 for their time and received payment after 1–2 weeks of the study.

Median participants in the study had a childhood household income between $75,000 and $100,000, current household income between $25,000 and $44,999, and reported saving 5–10% of their current income. Median participants were also college graduates, and 77% of participants reported having not experienced extended unemployment in the past 2 years. Participants were also asked to respond to financial knowledge questions^49^ probing their understanding of credit ratings and investments. On average, participants correctly answered 73%, or 11 of the 15 financial knowledge questions (*SD* = 2.4).

### Procedure

Our experimental design approach inherits heavily from prior publications adopting the SHOP paradigm^21,22,25^. To facilitate comparisons with benchmark SHOP studies, we retained the basic trial structure and added a payment method screen and two payment review screens (Fig. 1). The payment method screen (cash or credit card) was inserted between the product presentation and price screens. This sequencing was informed by results of a study showing that payment method matters if presented together with price information, but does not matter at the final checkout stage, after the consumer has presumably formed the intention to purchase^35^. The placement of the payment method prior to the price phase enabled us to examine whether the payment method modulated price-related or reward-related neural signals during the price differential computation. The trial ended with separate confirmation and checkout screens that required endorsement responses, giving participants a chance to “reflect on” but not change their decision, simulating the experience of receiving a receipt after a purchase. These additional stages were included to mimic the full sequence of a retail shopping experience and facilitate observation of post-decisional hedonics.

Each participant arrived to the study with their personal credit card and at least $50 in cash. Participants were told that they would be shopping within the lab’s experimental store, and that any purchases using cash or credit would be made through the lab at the end of the study. Therefore, any payments for purchases would come from a participant’s out-of-pocket funds rather than experimental endowments as in prior SHOP studies^21^. All products were offered at prices well below the minimum $50 cash on hand, with a median product price offer of $5.40 (*M* = $6.39, *SD* = $3.73, *min* = $1.50, *max* = $18.00). Similar to prior SHOP studies, these offered prices were at a fixed 70% discount relative to actual retail price (i.e., corresponding to retail prices between $5 to $60). Participants were required to bring at least $50 in cash to the study in order to minimize differential liquidity constraints; that is, participants did not reject items simply because they did not have enough cash with them, as we structured all products to have price offers to be below the $50 that participants had on hand. The prices examined within this study are at the high end in relation to previous literature applying the SHOP paradigm^21,22,25^ and behavioral research on payment method effects^41,41,50^. Yet, as we discuss within the conclusion, it is possible that other mechanisms could be at play when studying big-ticket items at prices higher than those examined in the current study.

To increase interest and simulate a typical retail experience, each participant faced a tailored set of product offerings. We populated a database of over 22,000 top selling items, drawing on product information from Amazon. An independent online sample then rated which categories they perceived to be most appealing, which reduced the database to approximately 4000 items, covering a wide range of categories, including beauty, kitchen, books, etc. Prior to entering the scanner, participants selected and rated the desirability of 42 categories from the lab’s experimental store on a 7-point scale. Products in personally more desirable categories were more likely to be offered in the fMRI shopping task.

The scanning task involved three shopping “runs,” with 28 trials each, or 84 in total. Within a trial, participants indicated whether they would buy a specific product at a stated price. If so, the product was added to the participant’s “shopping basket.” No products were repeated. Each product had a 50% chance of being offered for purchase with credit or with cash, pseudorandomly determined such that each payment method constituted half of the trials. At the end of the task, one product was randomly selected. If it was in the basket, the participant was asked to pay for the product at the stated price. Participants paid using their own personal credit card or out-of-pocket cash, as specified in the product offer. Regardless of payment method, items were shipped to participants by mail within 2–3 days of the study.

Each 24 s (s) trial consisted of six 4 s periods, followed by a jittered 2–8 s intertrial interval (see Fig. 1 for an illustration). Participants viewed a product in period 1; the payment method was introduced in period 2 with a cash or credit icon, the price in period 3. Participants signaled their decision to buy or not to buy in period 4. Following a buy decision, the participants saw a 4 s confirmation screen stating “this item has been added to your basket,” and a 4 s payment screen that required them to press a button to “commit to pay.” Following a no buy decision, the confirmation screen indicated “basket unchanged” and the payment screen required participants to press a button to acknowledge “no payment necessary.”

After exiting the scanner, participants reported their willingness to pay for each product shown in the scanner task by completing a separate incentive compatible auction procedure^51^ and also completed several psychological scales. Post-scan measures were not recorded for one participant due to a technical error.

### fMRI acquisition

All participants were right handed, native English speakers, with no history of neurological disorders. Participants were verified to have no magnetically reactive matter present in or on the body prior to scanning. All scans were performed using a 3 T Siemens Magnetom Tim Trio MRI System with a phase-array 32-channel head coil (Siemens Medical, Erlangen, Germany). Structural scans were acquired using a three-dimensional T1-weighted multi-echo MP-RAGE pulse sequence (TR = 2530 ms; TE = 1.64 ms, 3.5 ms, 5.36 ms, 7.22 ms; flip angle = 7°; slices = 176; thickness = 1 mm; matrix = 256 × 256). Task-based functional scans were collected using T2* weighted EPI sequence images sensitive to blood oxygen level-dependent (BOLD) contrast (TR = 2000 ms; TE = 30 ms; flip angle = 90°; slices = 32; thickness = 3 mm; matrix = 64 × 64). Analyses were conducted using the FMRIB Software Library, FSL, version 6.00^52^.

### Behavioral analysis

To model the effects of price and payment method on purchase decisions, we conducted a hierarchical logistic regression in which purchase decision was predicted by price, payment method, their interaction, and demographic controls. The hierarchical model included random slopes for the price × payment method interaction and participant-level random effects, following prior work^35^. Price was a continuous, z-normalized regressor, normed at the participant-level price distribution. The demographic variables (age, marital status, education level, and amount of savings) controlled for differences in shopping behavior across participants.

### ROI analysis

Region of interest analyses examined activity in a priori determined focal brain areas selected based on past observations that have isolated neural purchasing signals, as described above^21,22,25^. To specify the precise regions for analysis, we applied masks from meta-analyses of the striatum and VMPFC (see Fig. 9 within Bartra et al.^26^ for brain maps depicting these regions), as well as the rAIC^34^, k = 2, cluster 2. Notably, the striatum contains the nucleus accumbens, an ROI referred to in past research^21,22,25^. See Fig. 3 for a graphical display of the ROIs.

These meta-analytically determined brain regions match the ROIs examined in prior SHOP experiments while offering interpretive advantages through the application of sample-independent functional definitions rather than sample-dependent anatomical definitions. Furthermore, automated ROI selection served to minimize potential experimenter bias associated with the manual adjustment of ROI coordinates for individual participants. ROIs for the ventral striatum and VMPFC were generated based on a five-way conjunction analysis identifying regions of the brain carrying a monotonic, modality-independent subjective value signal on the basis of thousands of independent brain scans^26^. The right anterior insula ROI was determined by applying a task-evoked coactivation-based parcellation analysis with hundreds of independent scans^34^. Whole brain contrast analyses verified that striatum activation was associated with product preference, VMPFC activation was associated with choice, and right anterior insula activation was associated with higher prices within our sample (see Supplementary Information).

Prior findings in the SHOP paradigm established that differential neural purchasing signals emerge during the price and choice phases^21,22,25^. Thus, we anticipated that the payment method would impact these neural purchasing signals at the price and choice phases, following presentation of the payment method. We focus our analysis and interpretation on these stages of the time course in which payment methods were predicted to modulate neural purchasing signals (in addition to the payment method phase), but we also provide results at all other stages of the time course for the reader’s reference (that is, including stages prior to the presentation of payment method itself and stages after participants already recorded a purchase decision). Our goal was to understand how payment method influenced the previously identified ROIs when making purchase decisions. In order to present these effects intuitively, we report the results of logistic regressions conducted separately for each ROI and at each acquisition point. The figures report parameter significance from logistic regression results without corrections; please note that the key interaction effects in the striatum remain significant after Bonferroni corrections.

Specifically, within each region of interest, we analyzed the relationship between signal change and purchasing behavior at each acquisition point (TR). Following prior literature applying the SHOP paradigm^21,22,25^, time courses were lagged by 4 s to compensate for the delay in the hemodynamic response; the time courses depicted in the figures reflect this 4 s lag. To identify the differential purchase signal associated with credit versus cash purchases, we first conducted logistic regressions of the purchase decision on the ROI signal change, payment method, and their interaction at each acquisition point (results shown in Fig. 4). In specific, for each ROI and acquisition point, we fit the following regression equation: $Buy=logit({b}_{0}+{b}_{1}\,*\,ROIactivation+{b}_{2}\,*\,PaymentMethod+{b}_{3}\,*\,ROIactivation\,*\,PaymentMethod)$; Buy corresponds to the decision to purchase (Buy = 1, NoBuy = 0), ROIactivation refers to the activation in the particular ROI at the acquisition point on the trial, PaymentMethod refers to the contrast coded treatment (Credit = 1, Cash = − 1).

We next evaluated the relationship between ROI activity and purchase behavior by conducting logistic regressions of the purchase decision on the ROI signal change and its interaction with price (a continuous, z-normalized variable; results shown in Fig. 5). Specifically, for the price interaction analysis in Fig. 5 we fit the following regression equation:$Buy=logit({b}_{0}+{b}_{1}\,*\,ROIactivation+{b}_{2}\,*\,Price+{b}_{3}\,*\,ROIactivation\,*\,Price)$. These analyses allowed us to directly examine the effects of payment method on previously identified ROIs involved in making purchase decisions.

Notably, all regression results apply price as a continuous, z-normalized regressor, normed based on the participant-level price distribution. Participant price distributions had minimum offer prices that ranged from $1.50 to $1.96 across participants and maximum price values that ranged from $12.78 to $18.00. “High-price” and “low-price” categories were included for graphical displays only (i.e., Fig. 5) and were defined relative to the median of each participant’s price distribution; binary price variables were not used as regressors in any significance tests. Further details regarding whole brain analyses as well as additional participant characteristics are provided within the Supplementary Information.

## Supplementary Information

Supplementary Information 1.
