# Supplementary material for: Neural mechanisms of credit card spending
Source: Sci Rep. 2021 Feb 18;11:4070. doi: 10.1038/s41598-021-83488-3 (PMC7892835; doi:10.1038/s41598-021-83488-3)
Supplement: Supplementary file 1 — Supplementary Information 1. [file 41598_2021_83488_MOESM1_ESM.docx]

**Title:** Neural Mechanisms of Credit Card Spending

**Authors:** Sachin Banker^a,b,*^, Derek Dunfield^b^, Alex Huang^b^, Drazen Prelec^b,c,d,e^

**Affiliations:**

^a^ Eccles School of Business, University of Utah, Salt Lake City, UT 84112

^b^ Sloan Neuroeconomics Laboratory, Massachusetts Institute of Technology, Cambridge, MA 02142

^c^ Sloan School of Management, Massachusetts Institute of Technology, Cambridge, MA 02142

^d^ Department of Brain and Cognitive Sciences, Massachusetts Institute of Technology, Cambridge, MA 02142

^e^ Department of Economics, Massachusetts Institute of Technology, Cambridge, MA 02142

^*^ To whom correspondence should be addressed: sachin.banker@eccles.utah.edu

**Neural mechanisms of credit card spending**

**Supplementary Information**

**Table of Contents**

Purchase behavior. 3

Product price distributions. 3

Participant characteristics. 3

Self-reported credit card habits. 3

Spendthrifts and tightwads. 4

Risk taking 4

Future time perspective. 4

Whole brain localization analysis. 5

Figure S1. Additional trial structure stimuli. 9

Comparison to Knutson et al. (2007). 10

Payment method interactions with price. 11

Figure S2. Purchase vs. non-purchase timecourses by price level. 12

References 13

# Purchase behavior.

All participants indicated an interest in purchasing multiple products. The average participant chose to purchase 32% of the eighty-four products offered (*M* = 27.1 products, *SD* = 12.0). Price differential served as a measure of consumer surplus, and was determined by the participant’s willingness to pay for the product minus the offered price of the product during the scan as a percent of the offered price (Karmarkar et al., 2015; Knutson et al., 2007). Purchased products had a positive price differential on average (*M* = .30, *SD* = 1.71) and unpurchased products had a negative price differential on average (*M* = -.80, *SD* = .38, *t*(760) = 17.2, *p* < .001), confirming that participants indeed found purchased products to offer a greater economic surplus compared to unpurchased products.

# Product price distributions.

Median prices for participant-level price distributions ranged across participants from $3.78 to $7.56 compared to $5.40 overall. In addition, means ranged from $4.80 to $8.46 compared to $6.39 overall and standard deviations ranged across participants from $2.39 to $4.71 compared to $3.73 overall. Minimum price values ranged from $1.50 to $1.96 across participants and maximum price values ranged from $12.78 to $18.00.

# Participant characteristics.

Self-reported credit card habits. Median participants reported using one credit card regularly and not engaging in credit card misuse (e.g., keeping the balance near their maximum, paying off a balance with another card, missing payments, etc.). Sixteen participants used a Visa card, six used an American Express card, and four used a Mastercard. Notably, participants disagreed with the propositions that “[they are] less concerned with the price of the product when [they] use a credit card” (*M* = 2.19, *SD* = 1.33, *t*(25) = 3.10, *p* < .01 versus scale midpoint of 3) and that “[they are] more impulsive when [they] shop with credit cards” (*M* = 2.54, *SD* = 1.36, *t*(25) = 1.73, *p* = .097 versus scale midpoint of 3).

Spendthrifts and tightwads. Consumers vary in their spending habits, in part due to individual differences in anticipated pain of paying. Among participants in our study, six were classified as tightwads, three were classified as spendthrifts, and seventeen were classified as unconflicted by the ST-TW scale (Rick et al., 2008). There were no differences in the percent of purchases made with credit between these groups (*F*s < 1).

Risk taking**.** We examined risk taking and risk perception subscales of the DOSPERT (Blais & Weber, 2006). Average risk taking scores across participants displayed slight risk aversion (*M* = 3.48, *SD* = .63, *t*(25) = 4.24, *p* < .001 versus the scale midpoint of 4). Average risk perceptions were not significantly different versus the scale midpoint. Both subscales were not significantly correlated with the share of purchases made with credit.

Future time perspective. On average, participants were somewhat forward looking, as measured by the Future Time Perspective Scale (Lang & Carstensen, 2002), *M* = 5.18, *SD* = 1.14, *t*(25) = 5.27, *p* < .01, versus the scale midpoint of 4. Forward looking tendency was not significantly correlated with the percent of purchases made with credit.

# Whole brain localization analysis.

In order to conduct whole brain verification analyses, we estimated a generalized linear model of the blood oxygenation level dependent (BOLD) response. For each participant, fMRI data were modeled with the following independent variables for each of the three runs: (R1) individualized product preference regressors determined by willingness to pay for each item, implemented as a parametrically modulated indicator variable during the product phase, (R2) price regressors equal to the offered price of the product displayed during the trial, implemented as a parametrically modulated indicator variable during the price phase, (R3) indicator variable for the decision to purchase or not purchase on the product during the choice phase, coded +1 for purchase and -1 for non-purchase, (R4) indicator variable for post-purchase confirmation, coded +1 for a purchase confirmation and -1 for a non-purchase confirmation. In addition, the model included several control regressors: (R5) indicator for the onset of the method phase, (R6) indicator for the onset of the pay phase, and (R7-12) six motion regressors. Choice and pay response phase regressors (R3 and R6) were modeled using a boxcar function with durations equal to the participant’s response times in that trial. The regressors had onsets tied to the start of the corresponding trial phase with durations lasting throughout that phase of the trial (i.e., two TRs). Regressors R1-R6 were convolved with a canonical hemodynamic response function. We then calculated first-level single-subject contrasts: (1) regressor R1 versus baseline, (2) regressor R2 versus baseline, (3) regressor R3 versus baseline, and (4) regressor R4 versus baseline, and subsequently conducted a second-level mixed-effects analysis.

This whole brain analysis was conducted in order to corroborate that our observations of neural activity corresponded to behavioral constructs of interest. First, we verified that product preference, measured by willingness to pay for the item, was correlated with activity in the striatum. The striatum, including both the caudate nucleus and the nucleus accumbens structures, has consistently been implicated in the representation of reward and value; recent studies have demonstrated that this brain area responds preferentially to positive stimuli, during both decisional tasks as well as during the receipt of reward, and by both primary and secondary sources of reward (Bartra et al., 2013; Clithero & Rangel, 2013; Knutson et al., 2007; Knutson & Karmarkar, 2014; Levy & Glimcher, 2012; Rangel & Clithero, 2013). Whole brain analysis indeed revealed a significant correlation between activity in the striatum and preference, as predicted (see Table S1).

To verify that the signal observed in the rAIC was related to the pain of paying, we checked that higher product prices were correlated with greater activation in the region. Signal in the rAIC has been associated with the anticipation of pain and negative emotions, and thus has been interpreted as encoding monetary loss within SHOP paradigms (Calder et al., 2001; Coghill et al., 1994, 1999; Critchley et al., 2004; Knutson et al., 2007, 2008; Mazar et al., 2016; Paulus & Stein, 2006). Whole brain analysis indicated that the offered price was in fact significantly correlated with greater activity in the rAIC during the price phase, as predicted (see Table S1).

Furthermore, we confirmed that activity in the ventromedial prefrontal cortex was correlated with buying behavior. Signal in the VMPFC has previously been interpreted as representing decisional value in choice settings (Bartra et al., 2013; Karmarkar et al., 2015; Knutson et al., 2001; Knutson & Karmarkar, 2014; Levy & Glimcher, 2012; Rangel & Clithero, 2013). In line with predictions, a whole brain contrast during the choice phase revealed that greater VMPFC activity was observed in purchase decisions relative to non-purchase decisions. Table S1 presents all areas of the brain that were correlated with the decision to purchase during the choice phase.

Full statistical maps from these contrasts are available on NeuroVault: https://neurovault.org/collections/XFHJPVBP/. Together, these results establish that the changes in neural activity we observed were related to predicted behavioral constructs.

**Table S1.** Whole brain activation foci for preference, price, and purchase decisions (predicted regions in italic; Talairach Daemon Labels).

|  |  |  | **MNI Coordinates** | | |
| --- | --- | --- | --- | --- | --- |
|  | **Peak Z** | **Cluster Size (voxels)** | **Right**  **(x)** | **Anterior**  **(y)** | **Superior**  **(z)** |
| **Preference** |  |  |  |  |  |
| *Left caudate (incl. striatum)* | *5.29* | *49708* | *-8* | *10* | *0* |
|  |  |  |  |  |  |
| **Price** |  |  |  |  |  |
| Right parahippocampal gyrus (BA 19) | 4.85 | 5403 | 30 | -48 | -6 |
| Left occipital gyrus | 4.57 | 4215 | -34 | -86 | 8 |
| *Right cerebrum sub-lobar extra-nuclear (incl. right anterior insula)* | *3.78* | *430* | *32* | *22* | *0* |
| Right cingulate gyrus (BA 32) | 4.06 | 328 | 8 | 28 | 38 |
| Right frontal lobe Precentral gyrus | 3.6 | 236 | 44 | 12 | 34 |
| Right inferior parietal lobule (BA 40) | 3.38 | 186 | 46 | -40 | 44 |
| Right frontal lobe Precentral gyrus (BA 9) | 3.32 | 172 | 42 | 30 | 32 |
| Right superior frontal gyrus | 3.27 | 167 | 24 | 58 | 8 |

| **Purchase** |  |  |  |  |  |
| --- | --- | --- | --- | --- | --- |
| Left cerebellum | 4.78 | 3004 | -44 | -64 | -40 |
| Right inferior occipital gyrus (BA 18) | 4.64 | 2360 | 26 | -92 | -6 |
| *Anterior cingulate (incl. ventromedial prefrontal cortex)* | *4.01* | *1962* | *0* | *48* | *8* |
| Left cingulate gyrus (BA 31) | 4.80 | 1174 | -2 | -36 | 38 |
| Left frontal lobe sub-gyral | 4.17 | 1163 | -22 | 34 | 50 |
| Left inferior parietal lobule (BA 40) | 3.24 | 312 | -52 | -58 | 44 |
| Left inferior frontal gyrus | 3.64 | 193 | -42 | 6 | 32 |
| Right middle frontal gyrus | 3.57 | 188 | 30 | 24 | 56 |
| Left middle frontal gyrus | 3.66 | 167 | -36 | 36 | -12 |

# Figure S1. Additional trial structure stimuli.


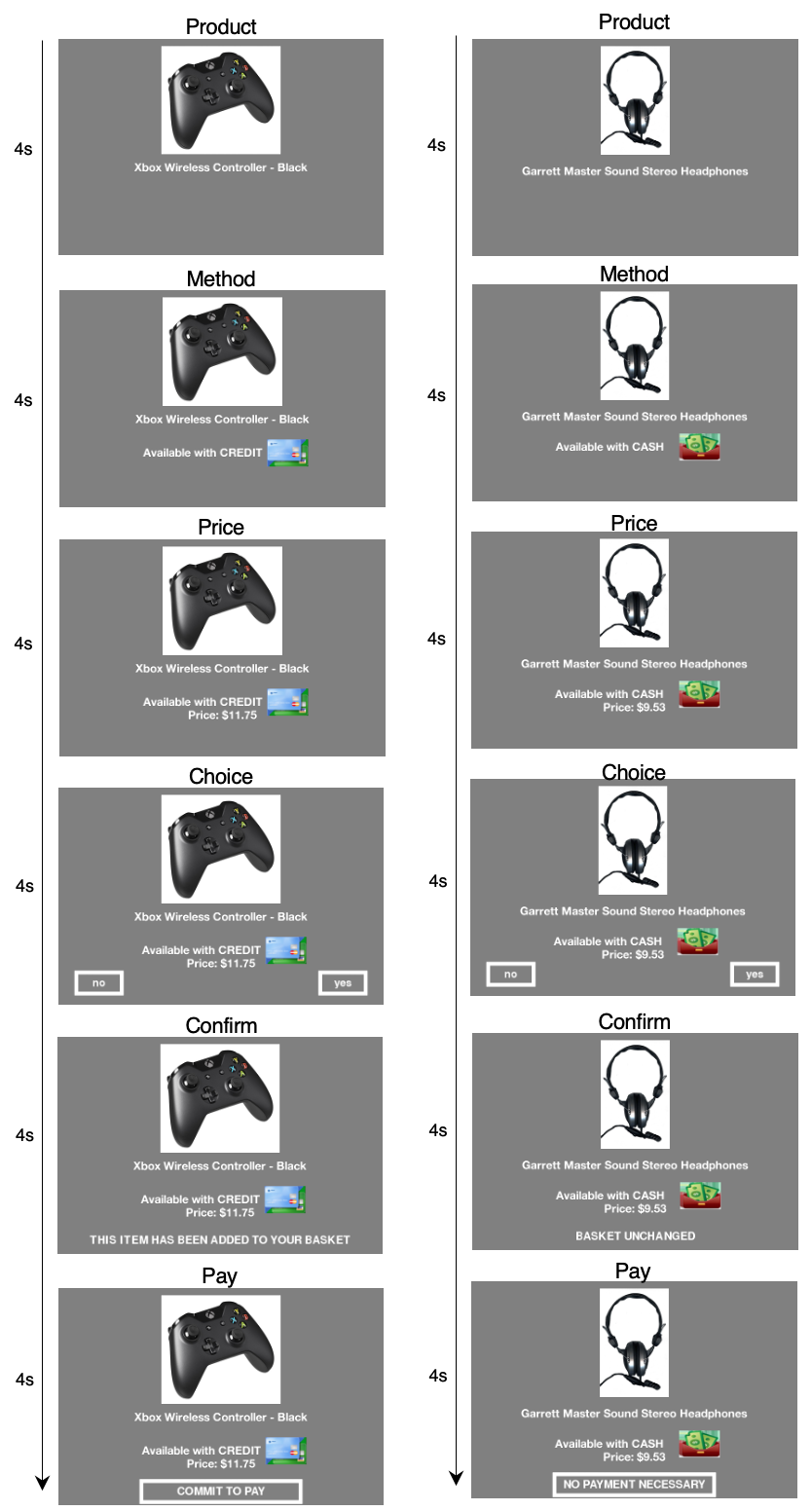


# Comparison to Knutson et al. (2007).

We examined the differential activation time courses in purchase and non-purchase trials within each ROI in the current study in order to offer a comparison to prior fMRI result from Knutson et al. (2007), in which the SHOP task was originally introduced (Knutson et al., 2007). All trials regardless of payment method and price were included in the analysis. Findings from the current study revealed a similar pattern of neural activation during shopping decisions as reported in Knutson et al. (2007); see Figure 2 in main text. We confirmed that purchase decisions were associated with increased activity in the striatum and VMPFC. However, we did not observe that purchasing was generally associated with deactivation in the rAIC, consistent with Karmarkar et al. (2015).

**Table S2.** Neural signals associated with purchase. The table below reports logistic regression results of the buy decision on each ROI signal intensity, payment method, and their interactions, at each TR. Red indicates a negative coefficient. Parameter significance denoted by *** p < .001, ** p < .01, * p < .05, ^ p < .10

|  | Product | | Method | | Price | | Choice | | Confirm | | Pay | |
| --- | --- | --- | --- | --- | --- | --- | --- | --- | --- | --- | --- | --- |
|  | 2s | 4s | 6s | 8s | 10s | 12s | 14s | 16s | 18s | 20s | 22s | 24s |
| Striatum |  |  | ^ | *** | *** | *** | *** | ** |  |  | ^ | ^ |
| VMPFC |  |  | ** | ^ |  |  |  |  |  | * | *** | *** |
| rAIC |  |  |  |  |  | ^ |  |  | ** | *** | ** | *** |
| Credit |  |  |  |  |  |  |  |  |  |  |  |  |
| Credit x Striatum |  |  |  | * | ** | *** | * |  |  |  |  |  |
| Credit x VMPFC |  |  |  |  |  |  |  |  |  |  |  |  |
| Credit x rAIC |  |  |  |  |  |  |  |  |  |  |  |  |

# Residual striatum activation analysis.

Supplementing the results reported within the main text, we conducted an additional analysis using residual striatum activation in which we partialled out the variance associated with the VMPFC and rAIC. To this end, we generated striatum activation residuals by regressing the striatum signal on VMPFC and rAIC signal at each time point. We then entered the residuals into the logistic regression model from Figure 4, replacing striatum activation. These regression results are summarized below.

**Table S3.** Buy decision regressed on residual striatum signal intensity, payment method, and interaction at each TR. Red indicates a negative coefficient. Parameter significance denoted by *** p < .001, ** p < .01, * p < .05, ^ p < .10. Phases: * = product, M = method, $ = price, ? = choice, C = confirm, P = pay.

|  | Product | | Method | | Price | | Choice | | Confirm | | Pay | |
| --- | --- | --- | --- | --- | --- | --- | --- | --- | --- | --- | --- | --- |
| **Striatum** | 2s | 4s | 6s | 8s | 10s | 12s | 14s | 16s | 18s | 20s | 22s | 24s |
| Striatum(res) |  |  | ^ | *** | *** | *** | *** | * |  |  | ^ | ^ |
| Credit |  |  |  |  |  |  |  |  |  |  |  |  |
| Striatum(res) x Credit |  |  |  | ** | * | *** | * |  |  |  |  |  |

# Payment method interactions with price.

Supplementing the results reported in Figure 5 within the main text, below we report regression results which include direct contrasts between payment methods. This analysis yielded positive coefficients on striatum × price × credit interactions during the price phase. Because the coefficient on price was large and negative, this pattern of findings suggests that shopping with credit may attenuate the negative effect of price on purchase decision via heightened striatum activation.

**Table S4.** Buy decision regressed on ROI signal intensity, price (continuous), payment method, and all interactions at each TR. Red indicates a negative coefficient. Parameter significance denoted by *** p < .001, ** p < .01, * p < .05, ^ p < .10

| **Y=Buy** | Product | | Method | | Price | | Choice | | Confirm | | Pay | |
| --- | --- | --- | --- | --- | --- | --- | --- | --- | --- | --- | --- | --- |
| **Striatum** | 2s | 4s | 6s | 8s | 10s | 12s | 14s | 16s | 18s | 20s | 22s | 24s |
| Striatum |  |  |  | *** | *** | *** | *** | ** | * |  |  |  |
| Price | ^ |  |  | ^ | ^ | ^ | ^ |  | ^ | ^ | ^ | ^ |
| Credit |  |  |  |  |  |  |  |  |  |  |  |  |
| Striatum x Price |  |  | ^ |  |  |  |  |  |  |  |  |  |
| Striatum x Credit |  | ^ | ^ |  |  | ** | ** | * |  |  |  |  |
| Price x Credit | * |  |  | * | ^ | ^ | * |  | ^ | * | ^ | * |
| Striatum x Price x Credit |  |  |  | ^ | * | * |  |  | ^ |  |  |  |
| **VMPFC** |  |  |  |  |  |  |  |  |  |  |  |  |
| VMPFC |  |  | * |  | *** | * | ^ | * |  |  | * |  |
| Price | ^ | ^ |  | ^ | ^ | ^ | ^ | ^ | ^ | ^ | ^ |  |
| Credit |  |  |  |  |  |  |  |  |  |  |  |  |
| VMPFC x Price |  |  |  | ^ |  |  |  |  |  |  |  |  |
| VMPFC x Credit |  |  |  | * |  |  |  |  |  |  | ^ |  |
| Price x Credit | ^ | * | ^ |  | ^ | * | * | * | ^ | ^ | ^ |  |
| VMPFC x Price x Credit |  |  |  |  | ** | ^ |  |  | * | ^ |  |  |
| **rAIC** |  |  |  |  |  |  |  |  |  |  |  |  |
| rAIC |  |  |  |  |  |  |  |  | ^ |  | * |  |
| Price | ^ | ^ |  | ^ | ^ | ^ | ^ | ^ |  | ** |  |  |
| Credit |  |  |  |  |  |  |  |  |  |  |  |  |
| rAIC x Price |  |  |  |  |  |  |  |  |  |  |  |  |
| rAIC x Credit |  |  |  |  |  |  |  |  |  |  |  |  |
| Price x Credit | ^ | ^ | ^ | * | * | * | * | * | * | ^ | * |  |
| rAIC x Price x Credit |  |  | ^ |  |  |  |  |  |  |  |  |  |

# Figure S2. Purchase vs. non-purchase timecourses by price level.

The timecourses shown below correspond to those depicted within Figure 5 in the main text. Whereas Figure 5 in the main text displays activation differential, average purchase vs. non-purchase activation, below we plot purchase and non-purchase timecourses separately along with corresponding standard errors at each acquisition point. Purchase trials are shown with the solid lines and non-purchase trials are shown with the dotted lines.

# References

Bartra, O., McGuire, J. T., & Kable, J. W. (2013). The valuation system: A coordinate-based meta-analysis of BOLD fMRI experiments examining neural correlates of subjective value. *Neuroimage*, *76*, 412–427.

Blais, A.-R., & Weber, E. U. (2006). A Domain-Specific Risk-Taking (DOSPERT) Scale for Adult Populations. *Judgment and Decision Making*, *1*(1), 33–47.

Calder, A. J., Lawrence, A. D., & Young, A. W. (2001). Neuropsychology of fear and loathing. *Nature Reviews Neuroscience*, *2*(5), 352–363.

Clithero, J. A., & Rangel, A. (2013). Informatic parcellation of the network involved in the computation of subjective value. *Social Cognitive and Affective Neuroscience*, *9*(9), 1289–1302.

Coghill, R. C., Sang, C. N., Maisog, J. M., & Iadarola, M. J. (1999). Pain intensity processing within the human brain: A bilateral, distributed mechanism. *Journal of Neurophysiology*, *82*(4), 1934–1943.

Coghill, R. C., Talbot, J. D., Evans, A. C., Meyer, E., Gjedde, A., Bushnell, M. C., & Duncan, G. H. (1994). Distributed processing of pain and vibration by the human brain. *Journal of Neuroscience*, *14*(7), 4095–4108.

Critchley, H. D., Wiens, S., Rotshtein, P., Öhman, A., & Dolan, R. J. (2004). Neural systems supporting interoceptive awareness. *Nature Neuroscience*, *7*(2), 189–195.

Karmarkar, U. R., Shiv, B., & Knutson, B. (2015). Cost Conscious? The Neural and Behavioral Impact of Price Primacy on Decision Making. *Journal of Marketing Research*, *52*(4), 467–481.

Knutson, B., Adams, C. M., Fong, G. W., & Hommer, D. (2001). Anticipation of increasing monetary reward selectively recruits nucleus accumbens. *J Neurosci*, *21*(16), RC159.

Knutson, B., & Karmarkar, U. (2014). Appetite, consumption, and choice in the human brain. *The Interdisciplinary Science of Consumption*, 163.

Knutson, B., Rick, S., Wimmer, G. E., Prelec, D., & Loewenstein, G. (2007). Neural predictors of purchases. *Neuron*, *53*(1), 147–156.

Knutson, B., Wimmer, G. E., Rick, S., Hollon, N. G., Prelec, D., & Loewenstein, G. (2008). Neural antecedents of the endowment effect. *Neuron*, *58*(5), 814–822.

Lang, F. R., & Carstensen, L. L. (2002). Time counts: Future time perspective, goals, and social relationships. *Psychology and Aging*, *17*(1), 125.

Levy, D. J., & Glimcher, P. W. (2012). The root of all value: A neural common currency for choice. *Current Opinion in Neurobiology*, *22*(6), 1027–1038.

Mazar, N., Plassmann, H., Robitaille, N., & Lindner, A. (2016). Pain of Paying? — A Metaphor Gone Literal: Evidence from Neural and Behavioral Science. *SSRN Working Paper*. https://papers.ssrn.com/sol3/papers.cfm?abstract_id=2901808

Paulus, M. P., & Stein, M. B. (2006). An insular view of anxiety. *Biological Psychiatry*, *60*(4), 383–387.

Perry, V. G. (2008). Is ignorance bliss? Consumer accuracy in judgments about credit ratings. *Journal of Consumer Affairs*, *42*(2), 189–205.

Rangel, A., & Clithero, J. A. (2013). The computation of stimulus values in simple choice. *Neuroeconomics: Decision Making and the Brain*, *2*, 125–147.

Rick, S. I., Cryder, C. E., & Loewenstein, G. (2008). Tightwads and spendthrifts. *Journal of Consumer Research*, *34*(6), 767–782.
